# Identification and estimation of bioactive constituents Negundoside, Berberine chloride, and Marmelosin by HPLC and HPTLC for development of quality control protocols for Ayurvedic medicated oil formulation

**DOI:** 10.1186/s43094-021-00322-3

**Published:** 2021-08-26

**Authors:** Ajay Kumar Meena, P. Rekha, Ayyam Perumal, R. Ilavarasan, Ravindra Singh, N. Srikant, K. S. Dhiman

**Affiliations:** 1 Regional Ayurveda Research Institute, Aamkho, Gwalior, Madhya Pradesh 474009 India; 2 Captain Srinivasa Murthy Central Ayurveda Research Institute, Arumbakkam, Chennai, Tamil Nadu 600106 India; 3 Central Council for Research in Ayurvedic Sciences, Ministry of AYUSH, Government of India, New Delhi, India

**Keywords:** Anu Taila, Medicated oil, Ayurveda, Nasya, HPTLC, HPLC

## Abstract

**Background:**

Anu Taila is an ancient medicated oil Ayurvedic preparation that is commonly used for nasya karma. It contains more than 25 herbs and goat milk as per the Ayurvedic Formulary of India (AFI). It strengthens the neck, shoulder, and chest muscles and improves the capacity of sense organs. It delays the aging process and reduces hair fall. Recent studies showed that it is also useful in COVID-19. In the current study, an attempt to develop quality control protocols and evaluate the standardization parameters like refractive index, iodine value, saponification value, peroxide value, acid value, rancidity, HPTLC fingerprint profile along with major bioactive compound and quantification of Berberine chloride, Negundoside, and Marmelosin by HPLC. Establishing quality protocol and standard parameters like physicochemical parameters and estimation of bioactive compounds of this preparation is significant for quality control.

**Results:**

In this study, HPTLC identifies bioactive chemical compounds like Berberine chloride, Marmelosin, Negundoside, glycyrrhizin, and para hydroxybenzoic acid (PHBA), Lupeol, Embelin, and Solasodine, which were present in the Anu Taila formulation. HPLC was used to estimate the bioactive marker compounds Negundoside, Berberine chloride, and Marmelosin were present in the Anu Taila formulation. The quantitative evaluation of Berberine chloride (0.0013%), Marmelosin (0.0366%), Negundoside (0.0086%) is present in Anu Taila formulation.

**Conclusion:**

The study reveals that sufficient quality control parameters were followed during the preparation of the formulation. Physicochemical analysis was carried out as per the guidelines of Ayurvedic Pharmacopeia of India. HPTLC and HPLC profiles generated in this particular study can be considered as a preliminary tool ascertaining the authenticity of Anu Taila.

## Background

Ayurveda is an ancient science of India and helps the human body to keep fit while providing cures from indigenous plants, animal and mineral origin products for various ailments [1]. Ayurveda is a complete and holistic traditional healthcare system of India that contains both preventive and therapeutic aspects [2, 3]. Medicated oil is one of the important dosage forms widely described in Ayurvedic pharmaceutics. For Taila preparation, specific oil is boiled with prescribed liquid media (Svarasa or Kwatha, etc.) and a fine paste (Kalka) of the drugs specified in the formulation composition. Unless specified otherwise, taila means Tila taila [4].

Anu Taila is an Ayurvedic medicated oil used for nasya since ancient times to cure dryness of the skin (twak raukshya), graying of hair (palita), a disorder of body parts above clavicle (urdh vajatrugatarog), emaciation of shoulder (skandha shushkata), wasting in cervical region (greeva shushkata), emaciation of chest muscles (vaksha shushkata) [4, 5]. Anu Taila contains about 25 herbs *Aegle marmelos* (roots), *Aquilaria agallocha* (heartwood), *Asparagus racemosus* (roots), *Berberis aristata* (stem), *Cedrus deodara* (heartwood), *Cinnamomum tamala* (leaves), *Cinnamomum zeylanicum* (stem bark), *Coleus vettiveroides* (roots)*, Cyperus scariosus* (rhizomes), *Cyperus rotundus* (rhizomes), *Desmodium gangetium* (whole plant), *Elettaria cardamomum* (seeds), *Embelia ribes* (fruits), *Glycyrrhiza glabra* (roots), *Hemidesmus indicus* (roots), *Leptadenia reticulata Linn.* (roots), *Nelumbo nucifera* (flowers), *Nymphaea stellata* (flowers), *Pluchea lanceolata* (roots), *Santalum album* (heartwood), *Solanum indicum* (whole plant), *Solanum surattense* (whole plant), *Uraria Picta* (whole plant), *Vetiveria zizanioides* (roots), *Vitex negundo* (seeds), water, goat milk, and all the ingredients are blended in the form of decoction as per the Ayurvedic Formulary of India (AFI) [4, 6]. This decoction is slowly infused with sesame oil (Tila taila) over a long time with controlled heating until the oil's desired quality is obtained. Goat milk is also used in the last cycle only. Hence, it is said that regular use of Anu Taila nasya regains the sharpness of the sense organs, clarity of voice, and facial glow. It strengthens the muscles of the neck, shoulders, and chest [7, 8]. It cures hair fall and prevents premature graying of hair and the premature appearance of wrinkles on the face [9]. In the recent clinical study, Anu Taila is used as one of the medicines in Ayurvedic treatment regime as nasal drop in the treatment of COVID-19.

Moreover, disease of the upper part of the body remains no more frequent with the regular use of Anu Taila. An Ayurvedic formulation must confirm test for identity, potency, purity, safety, and efficacy as per Pharmacopeial standards and WHO guidelines [10]. Quality assurance of traditional formulations relies upon good manufacturing practices with adequate batch-to-batch analysis and a standardized method of preparation [11].

In the current study, an attempt was made to develop quality control protocols and evaluate the standardization parameters like refractive index, iodine value, saponification value, peroxide value, acid value, rancidity, HPTLC fingerprint profile along with major bioactive compound and quantification of Berberine chloride, Negundoside, and Marmelosin by HPLC. Establishing quality protocol and standard parameters like physicochemical and other parameters and estimating this preparation's therapeutic bioactive compounds are highly significant for quality control. Routine use of such scientific techniques will lead to quality control and assurance of the Ayurvedic preparations to a certain extent. It would help in building confidence in the use of these Ayurvedic formulations [12]. We evaluated the Taila for physicochemical parameters, HPTLC fingerprint profiling, and HPLC quantification of selected bioactive markers.

## Methods

### Chemicals, reagents, and reference standards

The Anu Taila used in the present study was procured from the local market Chennai, Tamil Nadu, in packing of 100 ml (10 bottles). All chemicals and solvents used were of AR and HPLC grade. The reference standards of markers were procured from Natural remedies, Bengaluru, India.

#### Preparation of standard solution

Accurately weighed required quantity of Negundoside (3 mg), Berberine chloride (2.2 mg), Marmelosin (2 mg), glycyrrhizin, para hydroxybenzoic acid (PHBA), Lupeol, Embelin, and Solasodine (2 mg each) was dissolved in HPLC grade methanol in a 10-ml volumetric flask separately, sonicated for 10 min and finally made up to the marker with methanol to obtain standard stock solutions of 0.3 mg/ml Negundoside, 0.22 mg/ml Berberine chloride and 0.2 mg/ml Marmelosin, glycyrrhizin, para hydroxybenzoic acid (PHBA), Lupeol, Embelin, Solasodine, respectively. These solutions were used as standard stock solutions for HPTLC and HPLC studies.

#### Preparation of test solution

Five milliliters of Anu Taila was shaken with 10 ml of methanol. The mixture was allowed to stand till the two layers separated. The methanolic layer was separated, filtered through a 0.22-μ membrane filter, and used for HPTLC and HPLC analysis [4].

#### Physicochemical parameters

The Anu Taila was evaluated for physicochemical parameters, like refractive index, acid value, saponification value, iodine value, peroxide value, rancidity, and specific gravity carried out as per Indian Pharmacopoeia and Ayurvedic Pharmacopoeia of Indian standard methods [4, 13].

#### High-performance thin-layer chromatography (HPTLC) fingerprint profiling [10, 14–17]

Bioactive constituents and Anu Taila were subjected for HPTLC study using different solvent systems, and optimization was carried out for each marker and formulation. The solvent system was optimized to get the maximum separation of various phytochemicals, and it was further used for the HPTLC study.

Two microliters of Anu Taila test solution and 10 µl of each reference standard solution were applied on Tracks 1–9 on E. Merck Aluminum plate was pre-coated with silica gel 60F_254_ of 0.2-mm thickness using CAMAG Automatic sample applicator (ATS-IV). The plate was developed in the Twin trough TLC Chamber saturated with the solvent system of *toluene/ethyl acetate/chloroform/methanol/formic acid (8:0.5:0.5:0.2:0.2; v/v/v/v/v)* and dried. The developed plate was observed through CAMAG TLC Visualizer under UV at 254 nm and 366 nm. Photographs were documented and scanned using CAMAG TLC scanner with WINCATS software at a wavelength of UV at 254 nm and 366 nm using deuterium and mercury lamps, respectively. Finally, the plate was dipped in vanillin sulfuric acid reagent and heated in a hot air oven at 105 °C until the color of the spots appeared. The derivatized plate was photo-documented under white light and scanned using CAMAG TLC scanner with WINCATS software at a wavelength of 540 nm using a tungsten lamp.

#### High-performance liquid chromatography

The chromatographic evaluation was performed at room temperature using Agilent Technologies 1200 series and consisted of Agilent 1100/1200 Quaternary Pump, a manual sampler with 20 µL loop, and HPLC ChemStation 32 software. All samples and standards were filtered through 0.22-μ membrane filters. The chemical conditions have given below [18–21].

#### Chromatographic conditions for Negundoside

- *Instruments* Agilent 1200 series, manual sampler with VWD detector,
- *Column* Eclipse XBD C-18, 4.6 mm × 150 mm, 5 µm particle size,
- *Detection* VWD detector at 254 nm.
- *Injection volume* 10 μl.
- *Flow rate* 1 ml/min.
- *Temperature* 28 °C.
- *Retention time* 4.470.
- *Solvent system* Solution A: Solution B (15:85).
- *Solution A* Acetonitrile.
- *Solution B (Buffer)* Dissolve 0.136 g of anhydrous potassium dihydrogen orthophosphate (KH_2_PO_4_) in 900 ml of HPLC grade water, add 0.5 ml of orthophosphoric acid, and make up to 1000 mL with HPLC grade water, filter through 0.45-m membrane and degas for 3 min using a sonicator.

#### Chromatographic conditions for Berberine chloride

- *Instruments* Agilent 1200 series, manual sampler with VWD detector,
- *Column* Eclipse XBD C-18, 4.6 mm × 150 mm, 5 µm particle size,
- *Detection* VWD detector at 346 nm.
- *Injection volume* 10 μl.
- *Flow rate* 1 ml/min.
- *Temperature* 28 °C.
- *Retention time* 3.613.
- *Mobile phase* Gradient mixture of acetonitrile and phosphate buffer in following proportions.

**Table:** 

Time (min.)	Acetonitrile	Buffer
1	40	60
2	60	40
5	100	0
8	20	80
10	40	60
12	60	40
- *Solution A* Acetonitrile.
- *Solution B (Buffer)* Dissolve 0.136 g of anhydrous potassium dihydrogen orthophosphate (KH_2_PO_4_) in 900 ml of HPLC grade water, add 0.5 ml of orthophosphoric acid, make up to 1000 mL with HPLC grade water, filter through 0.45-m membrane, and degas for 3 min using a sonicator.

#### Chromatographic conditions for Marmelosin

- *Instruments* Agilent 1200 series, manual sampler with VWD detector,
- *Column* Eclipse XBD C-18, 4.6 mm × 150 mm, 5 µm particle size,
- *Detection* VWD Detector at 247 nm.
- *Injection volume* 10 μl.
- *Flow rate* 1 ml/min.
- *Temperature* 28 °C.
- *Retention time* 2.467.
- *Solvent system* Solution A: Solution B (80:20).
- *Solution A* Acetonitrile.
- *Solution B* Water.

## Results

### Physicochemical parameters

All the below mentioned physicochemical parameters of the Anu Taila were carried out as per the standard Indian Pharmacopeial and Ayurvedic Pharmacopoeia of India methods results, as tabulated in Table 1.

**Table 1:** Physicochemical parameter of Anu Taila formulation

S. no.	Test parameters	Results
1	Refractive index	1.47021
2	Acid value	8.36
3	Saponification value	211.48
4	Iodine value	101.80
5	Peroxide value	8.46
6	Rancidity	Positive
7	Specific gravity at 25 °C	1.0875

### High-Performance Thin-Layer Chromatography (HPTLC) fingerprint profile

The Anu Taila formulation was analyzed using an HPTLC fingerprint profile to confirm ingredients and bioactive constituents' presence. The R_f_ values and color of the spots are shown in Table 2.

**Table 2:** R_f_ values of various biomarkers and Anu Taila

S. no.	Tracks	At UV 254 nm		At UV 366 nm		After derivatization	
1		R_f_	Colour	R_f_	Colour	R_f_	Colour
2	Track 2: PHBA	0.08	Green	0.08	Blue	–	–
3	Track 3: Embelin	0.13	Green	0.13	Black	0.13	Yellow
4	Track 4: Marmelosin	0.41	Green	0.41	Blue	0.41	Yellowish gray
5	Track 1 and 5: Anu Taila	0.05	Green	0.05, 0.10 0.13, 0.24 0.35	Blue	0.04, 0.13	Violet
6		0.10, 0.13	Blue			0.18, 0.24 0.29, 0.32 0.38	Gray
7		0.18, 0.25, 0.35, 0.41, 0.45, 0.54, 0.61	Green			0.45, 0.48 0.54, 0.88	Violet
8	Track 6: Berberine chloride	–	–	–	–	0.24	Gray
9	Track 7: Solasodine	0.52	Green	–	–	0.52	Gray
10	Track 8: Lupeol	–	–	–	–	0.45	Violet
11	Track 9: Glycyrrhizin	–	–	0.57	Blue	–	–

### Quantitative estimation of Berberine chloride, Marmelosin, and Negundoside in Anu Taila formulation by HPLC

All the standards and Anu Taila test sample solutions were run in different mobile phase systems. Various proportions of the mobile phase were tried using water, acetonitrile, methanol, phosphate buffer, etc.

### Calibration curve

From the standard stock solutions of 0.3 mg/ml Negundoside, 0.22 mg/ml Berberine chloride, and 0.2 mg/ml Marmelosin, five further concentration dilutions were appropriately prepared with the same solvents, and the dilution concentrations are tabulated in Table 3. The calibration curve was established for peak area vs. concentration of Negundoside, Berberine chloride, and Marmelosin applied.

**Table 3:** Details of calibration concentration dilutions of different standards

S. no.	Name of standards	Amount (mg)	Serial concentrations (mg/ml)
1	Negundoside	3 mg	0.3, 0.15, 0.075, 0.0375, 0.01875
2	Berberine chloride	2.2 mg	0.11, 0.0825, 0.055, 0.0275
3	Marmelosin	2 mg	0.2, 0.1, 0.05, 0.025, 0.0125, 0.00625

The obtained percentage amount of each bioactive marker in the Anu Taila polyherbal formulation is tabulated in Table 4.

**Table 4:** Amount of Berberine chloride, Marmelosin, and Negundoside present in Anu Taila formulation

S. no.	Formulation	Constituents	Amount of constituents present in the formulation (mg/ml)	Percentage of constituents present in the formulation
1	Anu Taila extract	Negundoside	0.0433	0.0086
		Berberine chloride	0.0065	0.0013
		Marmelosin	0.1834	0.0366

## Discussion

The physicochemical parameters, HPTLC fingerprint profiling, and various bioactive constituents estimate major bioactive constituents like Berberine chloride, Marmelosin, and Negundoside by HPLC of Anu Taila formulation were carried out as per standard Pharmacopoeia methods for Ayurvedic formulations [4–6]. The current study attempts to qualitatively identify various bioactive constituents using HPTLC and quantitative estimation of specific bioactive constituents in medicated Ayurvedic oil formulation Anu Taila using the HPLC method.

**Fig. 1:**
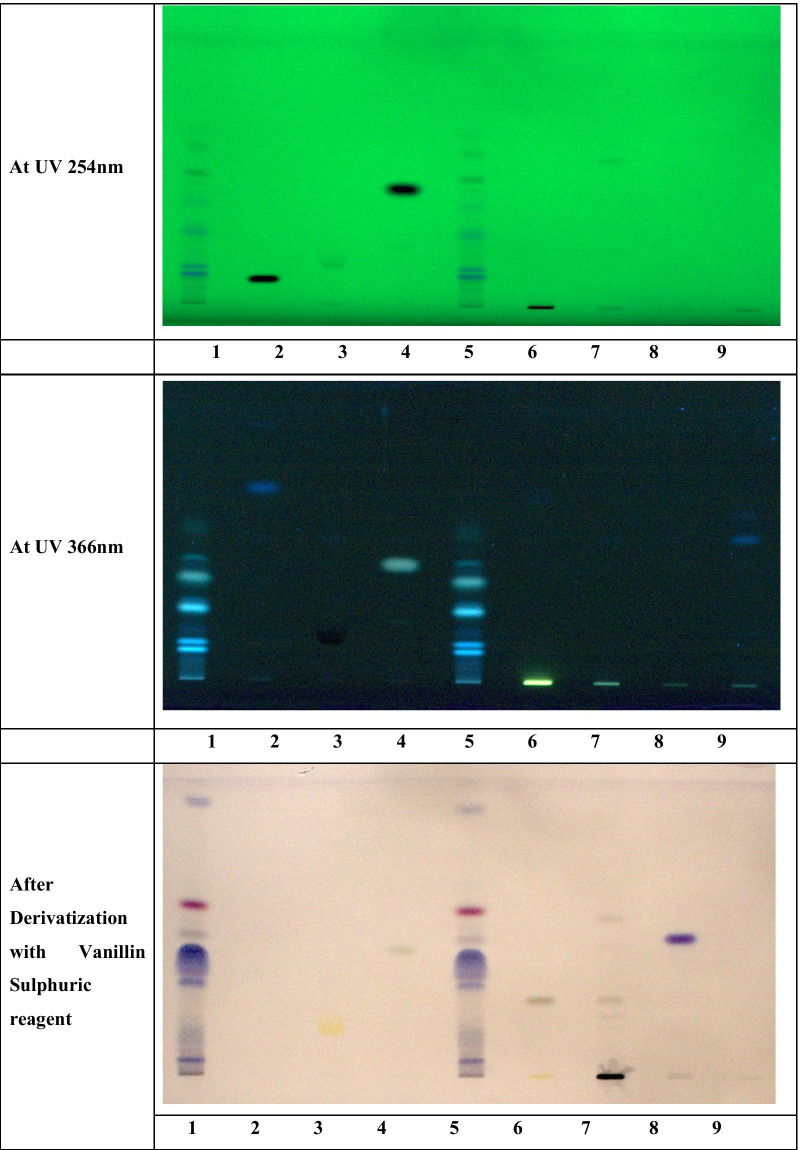
HPTLC fingerprint profile of methanol extract of Anu Taila and various biomarkers compounds. T1 and T5: Anu Taila, T2: PHBA, T3: Embelin, T4: Marmelosin, T6: Berberine chloride, T7: Solasodine, T8: Lupeol, and T9: glycyrrhizin. *Solvent system* toluene/ethyl acetate/chloroform/methanol/formic acid (8:0.5:0.5:0.2:0.2)

HPTLC profiling is an easy, low-cost, and specific method for identifying or qualitative and quantitative estimation of bioactive constituents [17]. For optimization, different mobile phase compositions were employed to achieve good separation. For analysis, some biomarker compounds, namely Berberine chloride, Marmelosin, Negundoside, glycyrrhizin, para hydroxybenzoic acid (PHBA), Lupeol, Embelin, and Solasodine, were selected for Anu Taila formulation, and the formulation is evaluated for the presence of major ingredients and therapeutic bioactive constituents. Developed HPTLC plates are shown in Fig. 1. The developed chromatogram has been included in supplementary Figs. 1, 2, and 3.

**Fig. 2:**
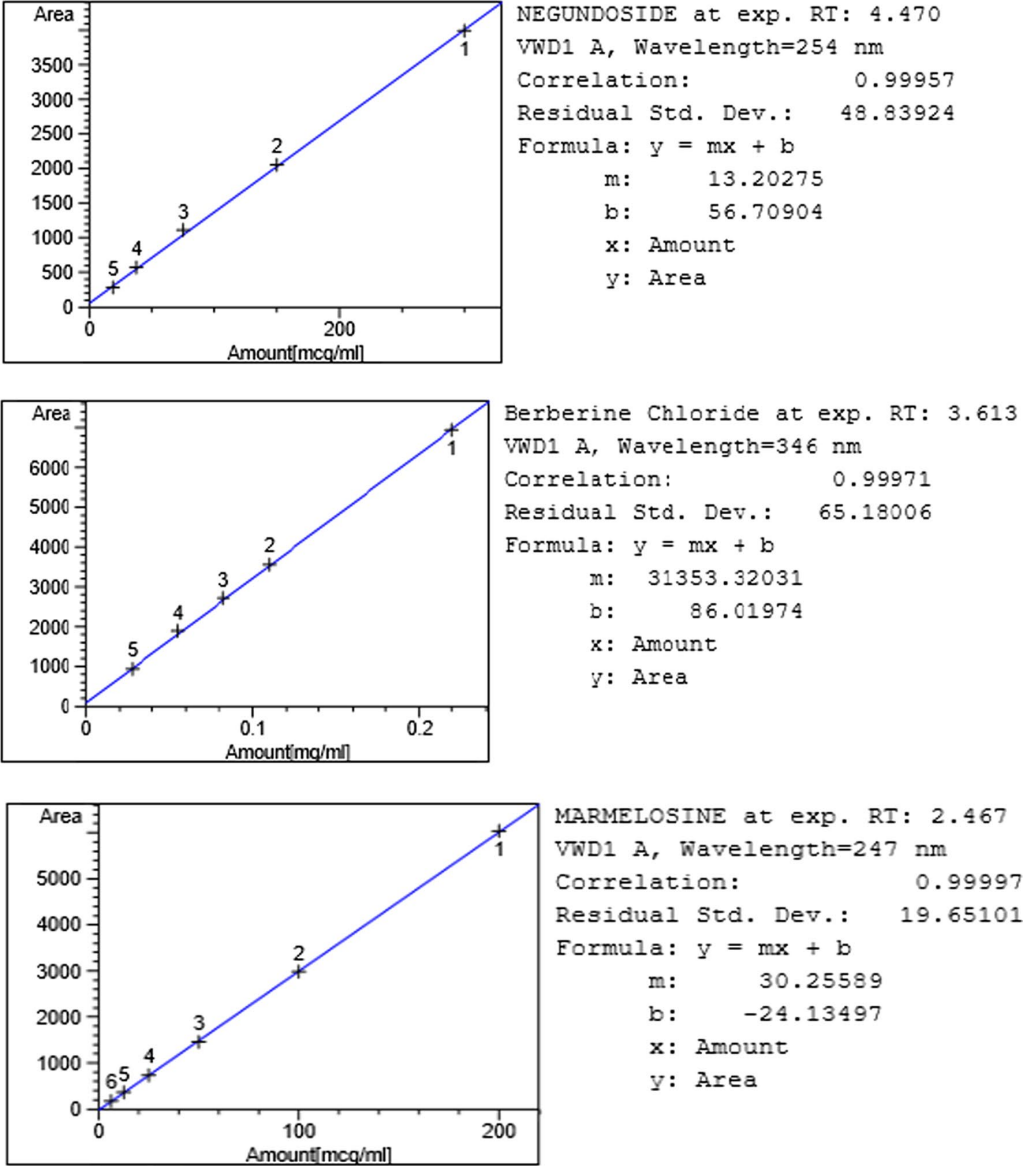
Calibration curve and regression coefficient of Negundoside, Berberine chloride, and Marmelosin

**Fig. 3:**
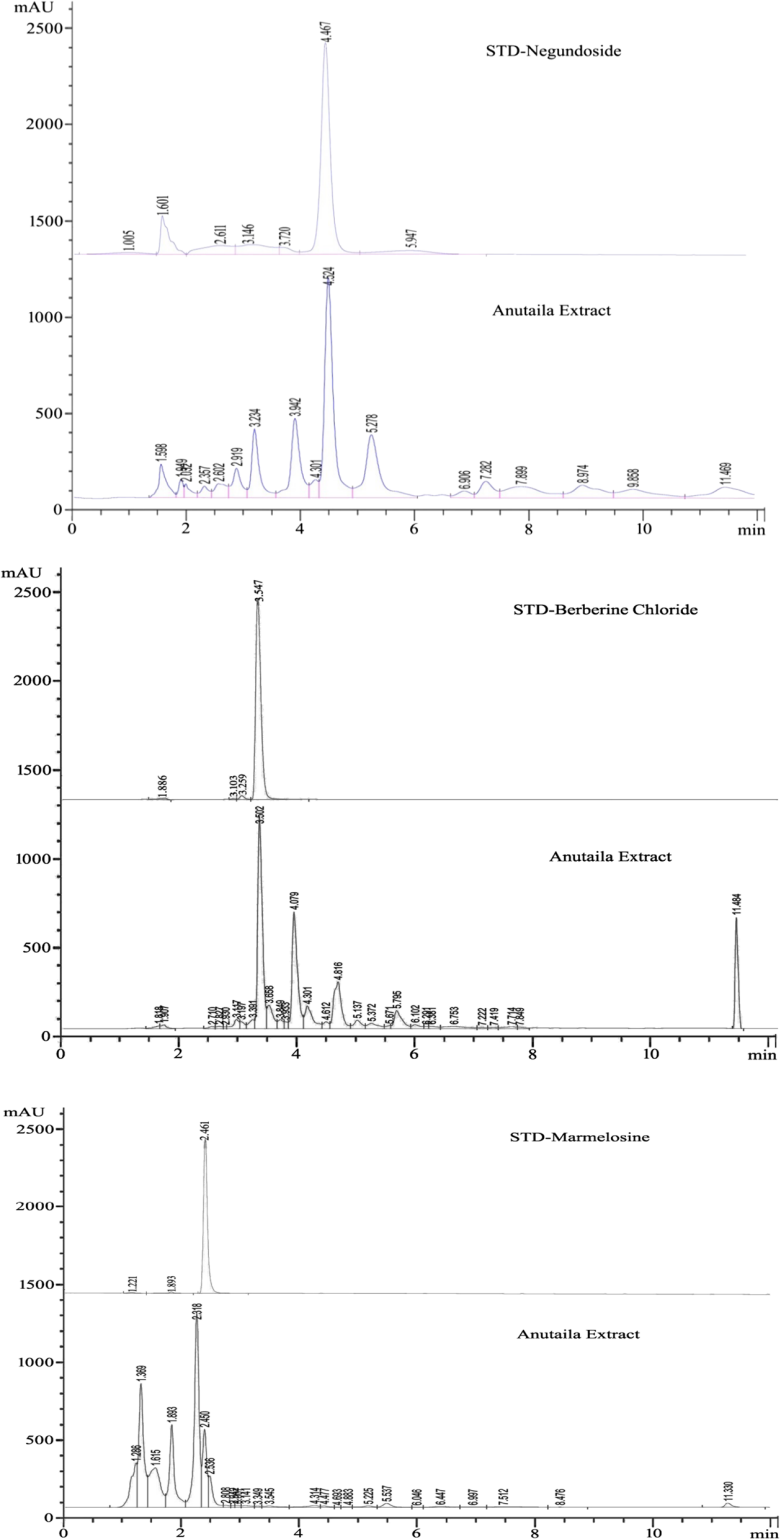
HPLC chromatogram of Negundoside, Marmelosin Berberine chloride standards, and Anu Taila formulation

A simple HPLC method, with isocratic elution, was developed for the qualitative and quantitative determination of Negundoside, Berberine chloride, and Marmelosin in Anu Taila Ayurvedic medicated oil formulation. A characteristic HPLC chromatogram was obtained using isocratic elution of the formulation extract, which exhibited a clean and smooth baseline with an excellent resolution where marker peak identified clearly. The marker compounds, namely Negundoside, Berberine chloride, and Marmelosin, are exhibited at the retention times 4.467, 3.547, and 2.461 for standard and 4.524, 3.502, and 2.450 for the formulation of the chromatogram. The linear calibration curve and regression coefficient analysis are given in Fig. 2.

The HPLC chromatograms of Berberine chloride, Marmelosin, Negundoside, and Anu Taila extract in the different mobile phases at different detection wavelengths, the retention time of various standards in Anu Taila as confirmed by the retention time of standard solution in the chromatogram at the same condition's details are given in Fig. 3.

These are the chemical constituents from one of the active ingredients of the Anu Taila and therefore useful for the quantitative estimation in quality control purpose.

## Conclusions

We standardized the Anu Taila an Ayurvedic formulation to evaluate various physicochemical parameters and HPTLC fingerprint profiles as per the Pharmacopeial standardization protocol of Ayurvedic medicated oil formulations. The development of chromatographic fingerprint profiles by the HPTLC method is an easy, accurate, economical, and specific method for identifying and quantifying biomarkers in formulations. In this study, HPTLC identifies some bioactive chemical compounds like Berberine chloride, Marmelosin, Negundoside, glycyrrhizin, and para hydroxybenzoic acid (PHBA), Lupeol, Embelin, and Solasodine, which were present in the Anu Taila formulation. HPLC was used to estimate the bioactive marker compounds Negundoside, Berberine chloride, and Marmelosin, which were present in the Anu Taila formulation. The quantitative evaluation of Berberine chloride (0.0013%), Marmelosin (0.0366%), Negundoside (0.0086%) was present in Anu Taila formulation. HPTLC and HPLC profiles generated in this particular study can be considered as a preliminary tool ascertaining the authenticity of Anu Taila.

## Data Availability

All data and materials are available upon request.

## Abbreviations

HPLC: High-performance liquid chromatography
HPTLC: High-performance thin-layer chromatography
PHBA: Para hydroxybenzoic acid
R_T_: Retention time
R_f_: Retention factor
TLC: Thin-layer chromatography
VWD: Variable wavelength detector
UV: Ultraviolet rays
WHO: World Health Organization
